# Enhancement and Homogenization of Indoor Air Quality in a Classroom Using a Vertical Airflow Ventilation Scheme

**DOI:** 10.3390/toxics10090545

**Published:** 2022-09-19

**Authors:** Su-Hoon Park, Kyung-Rae Lee, Se-Jin Yook, Hyun Bon Koo

**Affiliations:** 1 School of Mechanical Engineering, Hanyang University, Seoul 04763, Korea; 2 Department of Structural Engineering Research, Korea Institute of Civil Engineering and Building Technology, Goyang-si 10223, Gyeonggi-do, Korea

**Keywords:** classroom, ventilation, indoor air quality, age of air, computational fluid dynamics

## Abstract

Since air quality has a great influence on students’ health and learning ability, enhancing air quality in classrooms is important. Currently, widely distributed ventilation systems operate by moving airflow horizontally from ventilation inlets and outlets on the ceiling. This method can reduce the average pollution in a space by diluting it through air exchange; however, it is limited regarding homogeneous cleanliness due to air stagnation at some locations. Therefore, in this study, a new ventilation system was devised to improve indoor air quality and spatial homogeneity by installing ventilation inlets on the ceiling and numerous outlets on the floor, creating a vertical airflow in the interior space; this system was then applied to a middle school classroom. Using the age of air as an index, air quality improvement between the existing and newly designed ventilation systems was compared. In the classroom with the existing ventilation system, the age of air was low in the area near the ventilation inlets, while air congestion areas were widely distributed and air age was high near the outlets. Conversely, in the vertical airflow classroom, the average age of air was approximately 15% lower than that with the existing ventilation system, and the deviation of air age for each position in the classroom space was also reduced, showing a uniform air age distribution. Therefore, the vertical airflow ventilation system proposed in this study can be an effective ventilation scheme for enhancing and homogenizing indoor air quality.

## 1. Introduction

Although it is widely known that air pollution has a negative impact on human health, most breathing occurs indoors, and thus, efforts to identify and reduce the effects of exposure to indoor pollutants are important [1]. More harmful substances can be inhaled when working indoors, such as in offices and homes, than when working outside [2]. Therefore, indoor air quality management is especially important and is generally performed through ventilation. Maintaining appropriate indoor air quality by removing indoor pollutants through continuous ventilation helps increase work efficiency [3]. Furthermore, students with immature bodies should pay special attention to indoor air quality management because they take classes or study for a long time in an enclosed space such as a classroom. A previous study examined the effect of air quality improvement according to the type of ventilation system and ventilation rate on students’ academic achievement by measuring the concentration of various harmful substances such as carbon dioxide, formaldehyde, and fine dust in the classroom [4], and it is known that improving the air quality in a classroom greatly improves not only students’ health but also their learning ability [5]. As indoor air quality has a great influence on not only health but also productivity and academic achievement, several studies have been conducted regarding air quality improvement in various indoor spaces [6].

The age of air is often used as an indicator of air quality in research related to indoor air quality improvement. The age of air is used to evaluate ventilation efficiency based on the time it takes for fresh outside air to be supplied; the lower the age of air, the faster fresh outside air is supplied indoors, indicating a high ventilation efficiency [7]. Han [8] evaluated the ventilation efficiency of mechanical ventilation systems by calculating the age of air in empty spaces without obstacles through the numerical analysis method and reported that the local age of air in the space varies greatly depending on the indoor location. Li et al. [9] analyzed the airflow pattern according to the size of the space and the location of the ventilation inlets and outlets for the empty space where the mechanical vent was installed and reported that the airflow pattern varies depending on the location of the ventilation inlets and outlets, and there is a difference in local age of air in the space. Yang et al. [10] evaluated the indoor air quality of a bedroom with a wall-mounted air conditioner by obtaining the age of air through computational fluid dynamics (CFD) simulation, reporting that, when installing wall-mounted air conditioners, the age of air is acceptable when people are standing or sitting, but the age of air is poor when people are lying down. In addition, they found that CFD simulation can greatly aid in deriving a plan to maintain optimal air quality through CFD simulation because it can interpret various conditions. Ning et al. [11] evaluated indoor air quality according to the height of the ventilation inlets of an air conditioner placed on the bedside wall of the bedroom by obtaining the age of air through experiments and CFD simulations and confirmed that the age of air is improved by lowering the installation location of the ventilation inlets. Fischer et al. [12] conducted a study on the operation of a ventilation system installed in a classroom and claimed that intermittent operation of the ventilation system is not considerably helpful in improving indoor air quality, and, in the case of classrooms with poor ventilation, indoor air quality can be maintained comfortably only when ventilation is well performed at night. Jurelionis et al. [13] identified the fine dust removal efficiency for a space with one-way and four-way outlets installed and reported that the four-way outlet is more efficient in removing fine dust than the one-way outlet. Xing et al. [14] conducted a study on the improvement of indoor air quality by changing the location of ventilation inlets and outlets of a ventilation system in an empty indoor space without obstacles through CFD analysis and claimed that indoor air quality can be improved by up to 50% depending on the location of the ventilation inlets and outlets. Lin et al. [15] conducted a study on reducing pollutants in a general office space according to the location of the ventilation system through CFD analysis and reported that it is possible to achieve uniform air quality in the space when the location of the ventilation inlets is in the center rather than on the wall or on one side. Lim et al. [16] placed ventilation inlets and outlets of a ventilation system at various locations, confirming that the locations of ventilation inlets and outlets affects the reduction of harmful substances in the room, and presented guidelines for the arrangement of ventilation systems. Ahn et al. [17] evaluated indoor air quality by changing the arrangement of ventilation inlets and the ventilation scheme in an empty space divided with partitions and reported that the ventilation scheme and the arrangement of ventilation inlets significantly impact indoor air quality.

As discussed, previous studies have applied various ventilation schemes or changed the location of ventilation inlets and outlets to improve indoor air quality. However, the location of the ventilation inlets and outlets was limited to the ceiling or wall, and, to the best of our knowledge, there have been no studies on the formation of uniform air quality in an indoor space. However, in a space such as a classroom where many students remain in one space for an extended period, it is desirable to have a small deviation in the air quality, which affects the learner’s health and learning ability. To this end, it is necessary to uniformly distribute indoor air spatially. To improve indoor air quality as well as ensure spatial uniformity of air quality in a classroom, in this study, we applied a new structure in which the ventilation inlets are placed on the ceiling and the ventilation outlets are placed on the floor, apart from the typical configuration where the inlets and outlets of the ventilation system are both placed on the ceiling, and compared the distribution of age of air between existing and new types of ventilation inlet and outlet arrangements through numerical analysis and experiments.

## 2. Materials and Methods

### 2.1. Description of the Classroom

**Figure 1:**
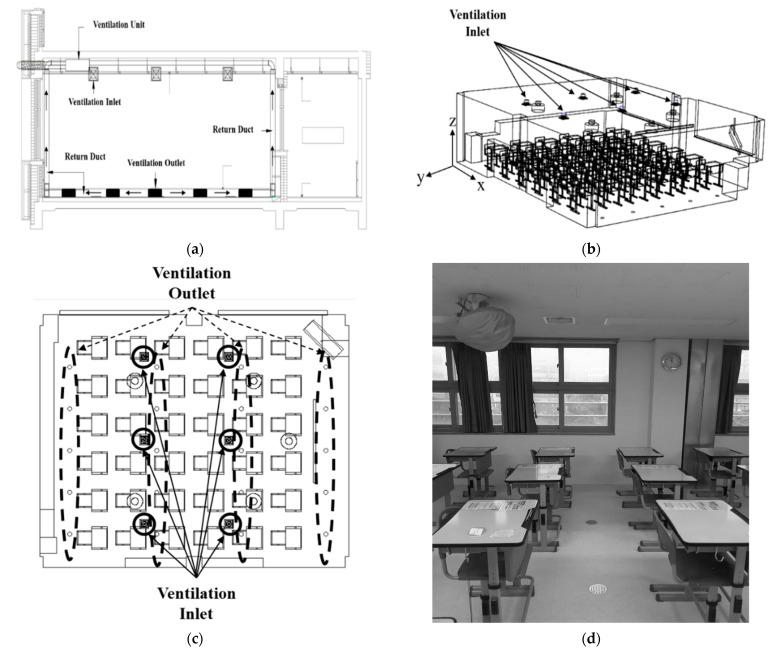
Vertical airflow ventilation of a classroom. (**a**) Schematic diagram of the ventilation system; (**b**) isometric view; (**c**) floor plan of the classroom as viewed from above; (**d**) photograph of the center of the classroom.

Figure 1a presents a schematic diagram of the newly devised ventilation system in which ventilation inlets were installed on the ceiling and ventilation outlets were installed on the floor to form a vertical airflow in the classroom. Figure 1b shows the simulation domain for the classroom newly devised in this study. The size of the classroom was 7.26 × 8.57 × 2.28 m. A total of 36 desks and chairs were arranged in a 6 × 6 configuration in the classroom. Figure 1c shows a floor plan of the classroom viewed from above; six ventilation inlets were installed in a 3 × 2 arrangement on the ceiling of the classroom, and 20 ventilation outlets were installed in a 5 × 4 arrangement on the floor of the classroom. Figure 1d is a photograph showing the center of the classroom, displaying the ventilation inlets on the ceiling and the ventilation outlets on the floor. As shown in the photograph, a fan was installed on the ceiling, but it was covered with a cover and not used.

**Figure 2:**
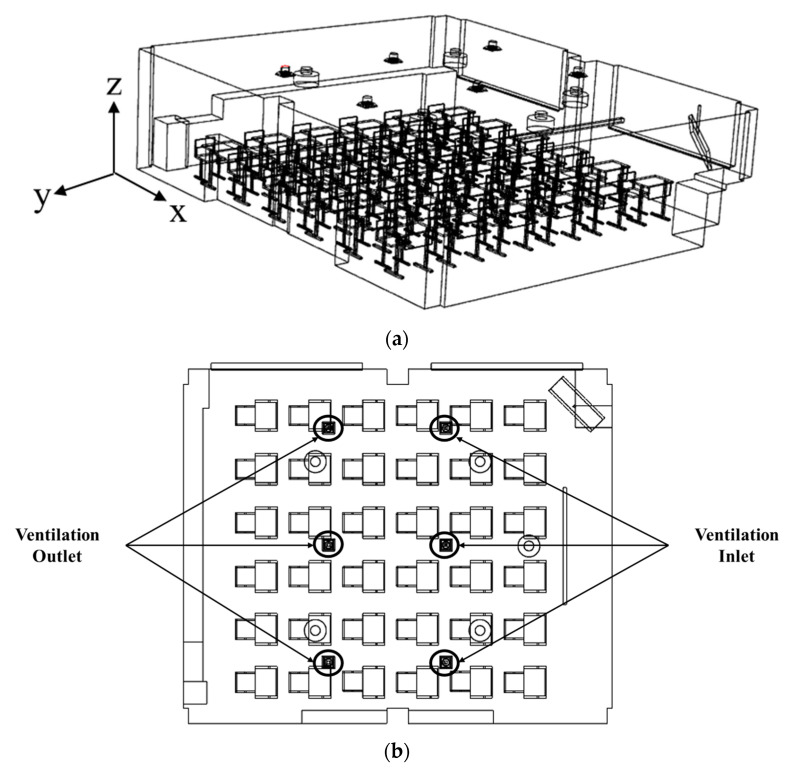
Reference classroom. (**a**) Isometric view; (**b**) floor plan.

Figure 2a shows the simulation domain for the reference classroom with the same size and desk-chair arrangement as those in the classroom shown in Figure 1. As presented in the floor plan in Figure 2b, three ventilation inlets were installed on the ceiling in front of the classroom and three ventilation outlets were placed on the ceiling in the back. Thus, the typical ventilation system structure was applied. The ventilation flow rate for the newly designed classroom in Figure 1 and the reference classroom in Figure 2 was set equally to 920 cubic meters per hour (CMH), which corresponds to 6.4 air changes per hour (ACH) for the volume of empty space in the classroom, and satisfied the recommended ventilation amount for a classroom space per the World Health Organization (WHO) (6 ACH) [18].

### 2.2. Numerical Method

ANSYS FLUENT *Release 18.0*, a commercial CFD code, was used to simulate airflow and age of air in the classroom. The flow was assumed to be three-dimensional, steady, incompressible, and turbulent. The standard *k-ε* turbulence model was used for turbulence analysis [19,20,21,22]. The governing equations for flow analysis were as follows [23]: 

Mass conservation equation:

(1) $$\frac{\partial \rho }{\partial t}+\nabla \cdot \left(\rho \vec{u}\right)=0$$

Momentum conservation equation:

(2) $$\frac{\partial \left(\rho \vec{u}\right)}{\partial t}+\nabla \cdot \left(\rho \vec{u}\vec{u}\right)=-\nabla p+\nabla \cdot \nabla \left(\mu \nabla \vec{u}\right)$$

Transport equation for *k* (standard *k-ε* model):

(3) $$\frac{\partial \left(\rho k\right)}{\partial t}+\nabla \cdot \left(\rho k\vec{u}\right)=\Delta \left[\left(\mu +\frac{\mu_{t}}{\sigma_{k}}\right)\nabla k\right]+G_{k}+G_{b}-\rho \varepsilon -Y_{M}$$

Transport equation for *ε* (standard *k-ε* model):

(4) $$\frac{\partial \left(\rho \varepsilon \right)}{\partial t}+\nabla \cdot \left(\rho \varepsilon \vec{u}\right)=\Delta \left[\left(\mu +\frac{\mu_{t}}{\sigma_{\varepsilon }}\right)\nabla \varepsilon \right]+C_{1\varepsilon }\frac{\varepsilon }{\kappa }\left(G_{\kappa }+C_{3\varepsilon }G_{b}\right)-C_{2\varepsilon }\rho \frac{\varepsilon^{2}}{\kappa }$$

**Figure 3:**
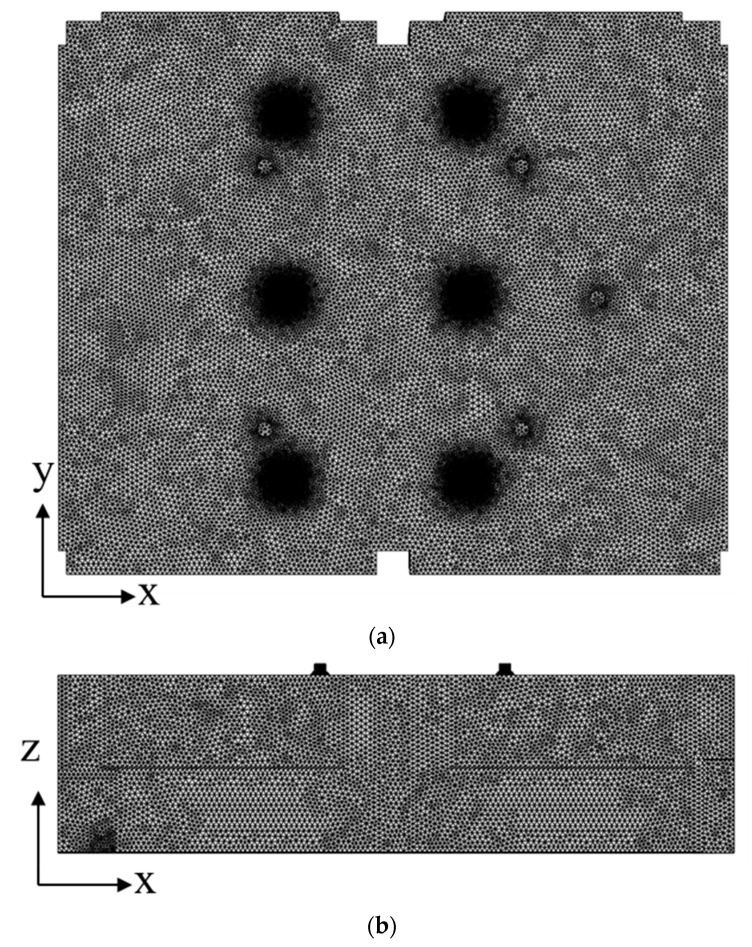
Grid configuration for a flow analysis of the classroom space along the (**a**) x–y plane and (**b**) x–z plane.

**Figure 4:**
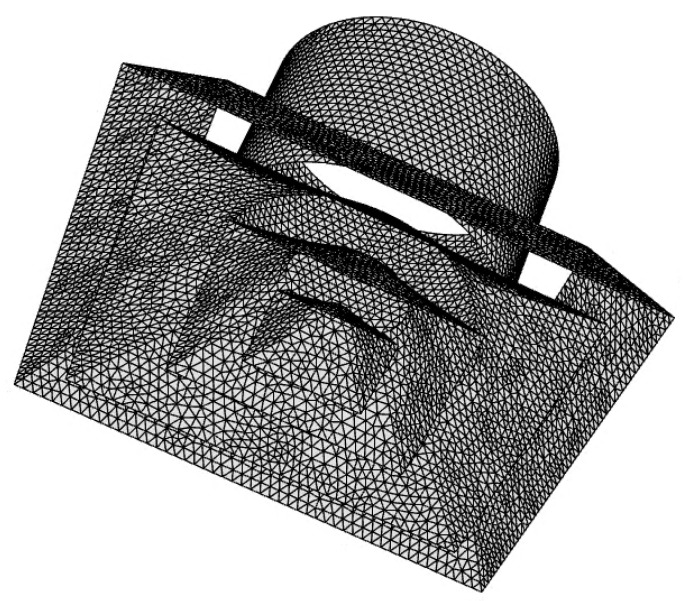
Grid configuration applied to a diffuser.

Along with flow analysis, the governing equations for analyzing the age of air using the User Defined Scalar were as follows [24,25,26]: 

Concentration Transport equation (age of air):

(5) $$\frac{\partial }{\partial x_{i}}\rho u_{i}\Phi -\dot{J}\frac{\partial \Phi }{\partial x_{i}}=\rho$$

(6) $$\dot{J}=-\left(\rho D_{m}+\rho D_{t}\right)\frac{\partial \Phi }{\partial x_{i}}$$

where *ρ* is the air density, *u_i_* is the flow rate, and Φ is the age of air. The diffusion term $\dot{J}$ is determined by the molecular diffusivity, *D_m_*, and turbulent diffusivity, *D_t_*. The air supply and exhaust flow rates of the ventilation system were set to 920 CMH. The indoor temperature and pressure were assumed to be 20 °C and 101.3 kPa, respectively. As boundary conditions for flow analysis, no-slip conditions were given for all walls in the classroom, velocity inlet conditions were given for ventilation inlets, and pressure outlet conditions were given for ventilation outlets. In particular, the same air supply flow rate was set in all ventilation inlets and the same exhaust flow rate was set in all ventilation outlets. In other words, the total ventilation flow rate of 920 CMH divided by the number of ventilation outlets was set to flow out from each ventilation outlet and the total ventilation flow rate of 920 CMH divided by the number of ventilation inlets was set to flow in through each inlet. For all equations that were solved, it was considered converged when the residual was less than 10^−4^. Figure 3 shows a mesh system generated using tetrahedral grids for classroom space. The grids near diffusers on the ceiling were more densely constructed, and Figure 4 presents the grid generated for each diffuser installed on the ceiling. The grid independency was checked by increasing the number of cells from approximately 5 million to 30 million. The average age of air at the height of 0.9 m was found to converge to a certain value, when the number of cells was more than 25 million. As a result, a mesh system with approximately 30 million cells was chosen for the simulation.

### 2.3. Experimental Method

An experiment was conducted to verify the prediction accuracy of the numerical analysis method. The experiment was conducted in a classroom of a middle school with the same configuration as that shown in Figure 1b, and a total of 36 desks and chairs were arranged in a 6×6 shape. A newly designed ventilation system was operated to form a vertical airflow as shown in Figure 1a, and the ventilation flow rate was set to 920 CMH. During the experiment, the temperature in the classroom ranged from 20 °C to 24 °C.

**Figure 5:**
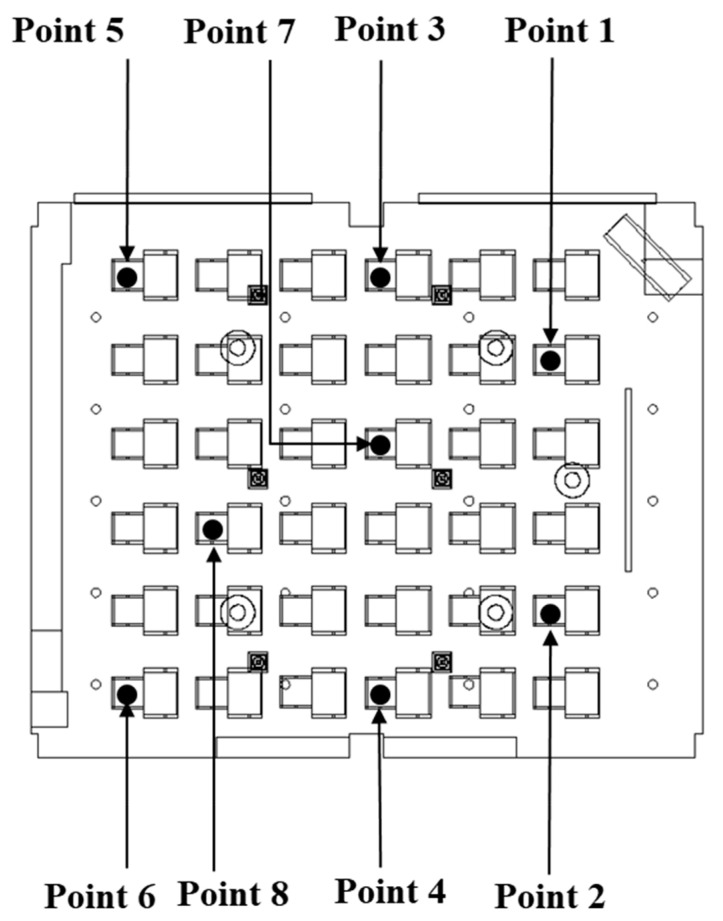
Particle number concentration measurement locations in the classroom.

The age of air was calculated through a change in the particle number concentration in the classroom. Particle number concentration in the air over time was measured using Optical Particle Counters (OPC; Model 1.108, Grimm Co., Ltd., Ainring, Bayern, Germany) at eight locations, as shown in Figure 5. As the sampling flow rate of each OPC was 1.2 L/min, it was assumed that its effect on the indoor airflow was negligible. Assuming that a 13-year-old middle school student in Korea breathes while sitting on a chair in a class, a sampling probe for inhaling aerosol was installed vertically at 0.9 m from the bottom of the classroom [25]. The experimental procedure for measuring age of air in this study was as follows [25,26]:

1. Leave all windows and doors open to allow outdoor air to enter with the ventilation system turned off (90 min);
2. Close all windows and doors, burn incense to generate particles, and turn on a fan to ensure that the particles spread evenly indoors (20 min);
3. [Step A] Turn off the fan and stabilize the flow of indoor air (20 min);
4. [Step B] Turn on the ventilation system and stabilize the flow of indoor air (10 min);
5. [Step C] Maintain a constant flow rate in the ventilation system (around 60 min).

**Figure 6:**
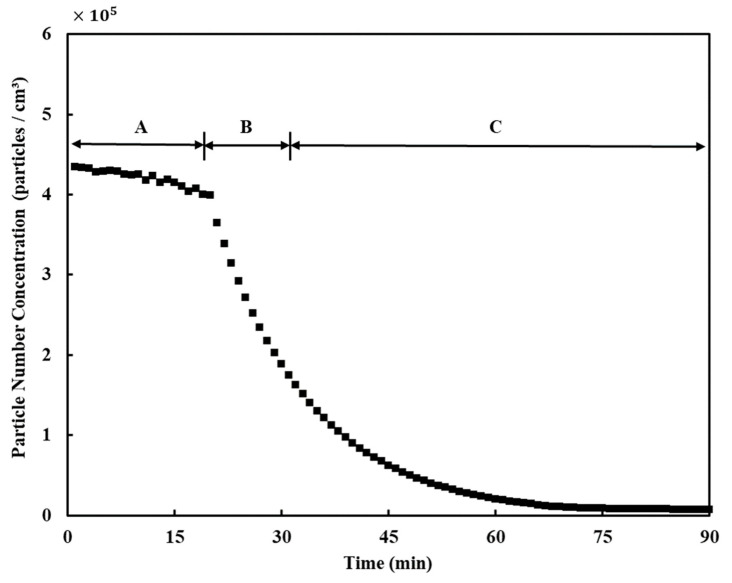
Example of particle number concentration measurement results over time.

Figure 6 shows an example of the results of the particle number concentration measurement of any OPC during the experiment according to the aforementioned procedure. During Step A, the particle number concentration decreased slowly, and, during Steps B and C, where the ventilation system began to operate, indoor air was exhausted, and particle-free air was supplied from the ventilation system, the particle number concentration decreased exponentially. Although there were differences in the reduction rate for each case and each measurement position, the particle number concentration decreased exponentially in all cases. Therefore, the age of air was calculated by an experimental method using the following equation [24,26]:

(7) $$C_{P}\left(t\right)=A\;\exp\left(-\frac{t}{\tau }\right)+y_{0}$$

where *A* is the initial particle number concentration at the beginning of Step C, *t* is the time, *y*_0_ is the value at which the particle number concentration converges after an extended period, and *τ* is the age of air obtained by curve fitting the experimental results. For reference, the derivation of Equation (7) can be found in Noh et al. [24].

## 3. Results and Discussion

**Figure 7:**
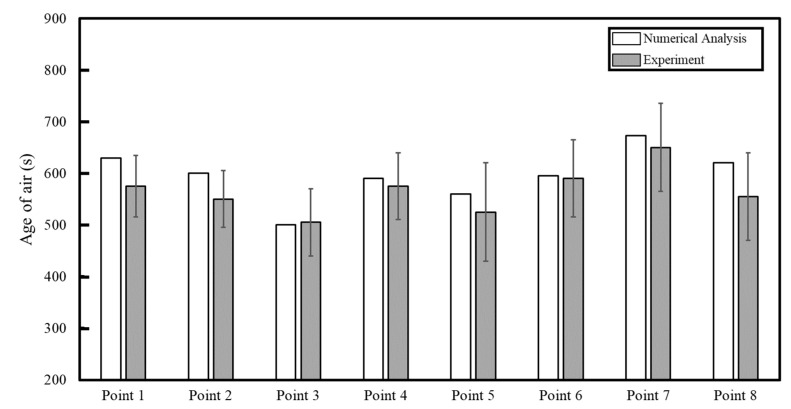
Validation of simulation results against the experimental results for the vertical air classroom.

Figure 7 shows a comparison of the age of air at eight locations in the classroom between the simulation and the experiment conducted on the vertical airflow classroom devised in this study. The ventilation flow rate through the six ventilation inlets on the ceiling and the 20 ventilation outlets on the floor was 920 CMH. The experimental results measured slightly lower than the simulation results at all other measurement positions except for Point 3; one of the reasons for this might be the fact that the particle deposition on indoor walls was not considered in the simulation whereas particles in the experiment could deposit on the walls owing to Brownian diffusion, turbulent diffusion, gravitational settling, etc. However, the tendency of the overall age of air distribution was well matched with the error of less than 10%. In case of the reference classroom, only the simulation was conducted, because the real reference classroom was not constructed in this study. Meanwhile, the simulation method of this study was also employed by Lee et al. [27,28], who investigated the age of air in a four-bed hospital ward in which both ventilation inlets and outlets were installed on the ceiling, like the reference classroom. According to Lee et al. [27,28], the simulation results agreed with the experimental data with the error of less than 10%. Therefore, the results of predicting the distributions of age of air in the vertical airflow classroom and the reference classroom using the numerical analysis method in this study were judged to be valid. Based on this, age of air simulation for the reference classroom shown in Figure 2 was also conducted to compare and analyze indoor air quality according to the ventilation scheme. For reference, the ventilation flow rate of the reference classroom was the same as that of the vertical airflow classroom (920 CMH), but the reference classroom had a typical vent arrangement with all three ventilation inlets and all three ventilation outlets installed on the ceiling.

**Figure 8:**
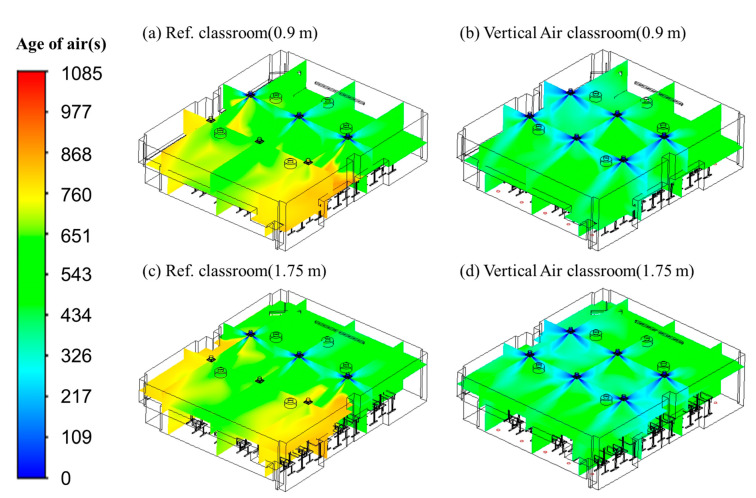
Age of air distribution. (**a**) Reference classroom, 0.9 m; (**b**) vertical air classroom, 0.9 m; (**c**) reference classroom, 1.75 m; (**d**) vertical air classroom, 1.75 m.

Figure 8 presents a comparison of the age of air distribution between the typical airflow configuration of the reference classroom and the vertical airflow classroom devised in this study when the ventilation flow rate was 920 CMH. A vertical observation surface was placed where the ventilation inlets and outlets were located, and horizontal observation surfaces were placed at a height of 0.9 and 1.75 m from the floor. As shown in Figure 8a,c, both the ventilation inlets and outlets were arranged on the ceiling in the reference classroom. The overall age of air was typically low in the front of the classroom where the three ventilation inlets were located due to the inflow of clean air, while the age of air was generally high in the back of the classroom where the three ventilation outlets were located. This is because the air introduced into the classroom through the ventilation inlets stagnated as it hovered in the space at the back of the classroom in the process of being sucked into the ventilation outlets. Thus, students in areas with high age of air in the classroom may face additional exposure to harmful substances than students in other locations because harmful substances are not smoothly exhausted and remain in a stagnant space for an extended period. As such, it was confirmed that if both the ventilation inlets and outlets were located on the ceiling, the difference in air quality around the ventilation inlets and outlets would be greatly distributed.

In contrast, in the case of the vertical airflow classroom as shown in Figure 8b,d, the age of air was found to be relatively evenly distributed in the classroom, and, although there were areas where the age of air was slightly higher than the average age of air, the range of these areas was relatively narrow. This is believed to be due to the considerable reduction in the area where air hovered and stagnated in the process of discharging clean air from the six ventilation inlets located on the ceiling through 20 ventilation outlets installed in various places on the floor. Moreover, when comparing the average age of air for the total volume of the classroom space, the vertical airflow classroom showed 589 s, which was 15% lower than that for the reference classroom (696 s). When comparing the age of air in different regions in the classroom by dividing the age of air in the front and back of the classroom with respect to the columns located on both sides of the classroom, the difference in average age of air between the front and rear parts of the classroom was 18% in the reference classroom, but was considerably lower at 1.5% in the vertical airflow classroom. Therefore, it was confirmed that the ventilation system in the vertical airflow classroom substantially reduces the airflow congestion section, improving indoor air quality and minimizing the bias of age of air distribution.

**Figure 9:**
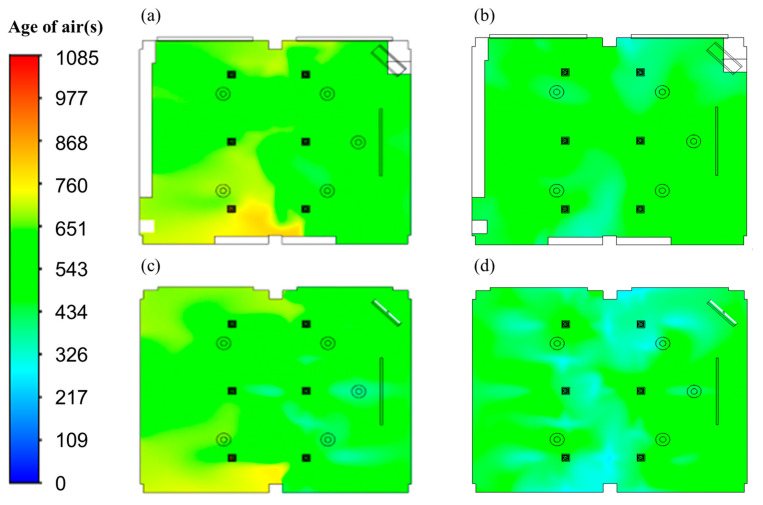
Contours of the age of air. (**a**) Reference classroom, 0.9 m; (**b**) vertical air classroom, 0.9 m; (**c**) reference classroom, 1.75 m; (**d**) vertical air classroom, 1.75 m.

Figure 9a,b show the age of air distribution in the reference and vertical airflow classrooms, respectively, at a height of 0.9 m from the floor, and this height considers the range of respiratory positions when students sit down during a class. The distribution of the age of air was more uniform in the vertical airflow classroom compared to that in the reference classroom. In addition, the average age of air at a height of 0.9 m was 720 s in the reference classroom and 603 s in the vertical airflow classroom; the age of air in the vertical airflow classroom was approximately 16% lower than that in the reference classroom. Figure 9c,d show the distribution of the age of air in the reference and vertical airflow classrooms, respectively, at a height of 1.75 m from the floor, and this height considers the range of respiratory positions when students stand up in the classroom. The average age of air at that height was 694 and 569 s in the reference and vertical airflow classrooms, respectively. Similar to that at 0.9 m height, the age of air in the vertical airflow classroom was approximately 18% lower than that in the reference classroom but was more uniformly distributed at a height of 1.75 m. For each type of classroom, the average age of air at a height of 1.75 m was lower than that at 0.9 m because the 1.75 m height was closer to the ventilation inlets on the ceiling, and hence, clean air typically reached this area faster.

**Figure 10:**
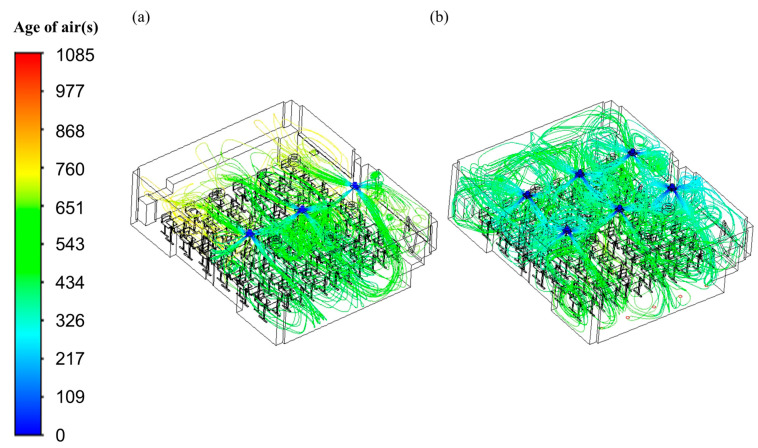
Path lines of airflow in the classroom. (**a**) Reference classroom; (**b**) vertical air classroom.

Figure 10 shows the path lines of airflow in the reference and vertical airflow classrooms, and the color of the path lines indicates the age of air at the corresponding location. As presented in Figure 10a, in the reference classroom, the clean air discharged from the ventilation inlet, moved down along the wall in the front of the classroom, and flew to the back of the classroom. Clean airflow was relatively smooth in the front of the classroom where the three ventilation inlets were located, resulting in a low age of air; however, in the back of the classroom, where the three ventilation outlets were located, the clean air supply was not smooth and stagnant areas were generated, forming a high age of air. Furthermore, as shown in Figure 10b, in the vertical airflow classroom, clean air discharged from the six ventilation inlets was exhausted through the 20 ventilation outlets installed over a large area of the floor; compared to the reference classroom, there were few areas in which airflow was stagnant, and clean air was smoothly supplied throughout the entire classroom. Through these differences in airflow patterns, it is possible to identify the reason why the vertical airflow classroom had improved indoor air quality and substantially reduced deviation according to location compared with those in the reference classroom.

**Figure 11:**
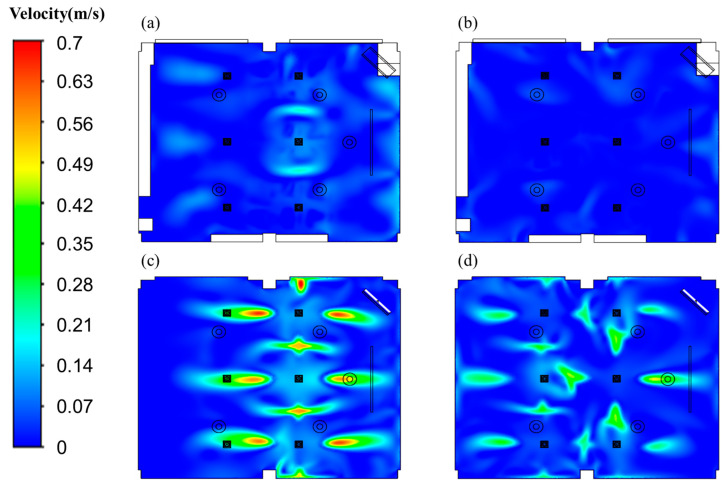
Contours of the air velocity. (**a**) Reference classroom, 0.9 m; (**b**) vertical air classroom, 0.9 m; (**c**) reference classroom, 1.75 m; (**d**) vertical air classroom, 1.75 m.

Figure 11a,b show the air velocity distribution in the reference and vertical airflow classrooms, respectively, at a height of 0.9 m from the floor. The air velocities were lower than 0.3 m/s with the average of 0.065 m/s in the case of the reference classroom, while those were less than 0.15 m/s with the average of 0.044 m/s in the case of the vertical airflow classroom. Figure 11c,d display the air velocity distribution in the reference and vertical airflow classrooms, respectively, at a height of 1.75 m from the floor. The maximum and average velocities at 1. 75 m height in the reference classroom were about 0.7 m/s and 0.1 m/s, respectively, while those in the vertical airflow classroom were approximately 0.5 m/s and 0.083 m/s, respectively. Even though the ventilation flow rates of both classrooms were equal, the vertical airflow classroom showed more uniform velocity distribution with lower average velocity. Therefore, the vertical airflow classroom is expected to be more favorable in terms of the local thermal comfort.

## 4. Conclusions

In this study, a new ventilation scheme was devised to create a vertical airflow by installing 6 ventilation inlets on the ceiling and 20 air ventilation outlets on the floor in a classroom of a middle school. The indoor air quality was then compared with that in a classroom to which the existing standard ventilation scheme was applied, where both ventilation inlets and outlets were installed on the ceiling. In both the vertical airflow classroom devised in this study and the existing ventilation classroom, the ventilation flow rate was set to 920 CMH, which corresponds to 6.4 ACH, satisfying the ventilation recommended for a classroom space by the WHO (6 ACH). The flow of air in the classroom was predicted through CFD simulation, and the age of air was analyzed using User Defined Scalar. Moreover, the age of air was calculated at eight places in the classroom via an experimental method by burning incense in the classroom to which the vertical airflow ventilation scheme was applied to generate particles and by measuring the particle number concentration decay due to the operation of the ventilation system. Based on the comparison of the age of air between the simulation and experiment, the age of air measured through the experiment was slightly lower than that derived from the simulation; however, the tendency was well-matched. Through this, the prediction accuracy of the simulation method used in this study was verified, and, based on this, the age of air was predicted in the reference classroom, where three ventilation inlets and three ventilation outlets were installed on the ceiling. In the reference classroom, the age of air was generally low in the front of the classroom near the three ventilation inlets, but in the back of the classroom near the three ventilation outlets, the age of air was high as the supply of clean air was not smooth and airflow was stagnated widely. Thus, the average age of air between the front and rear areas of the classroom showed a deviation of approximately 18%. In contrast, in the vertical airflow classroom designed in this study, clean air supplied through six ventilation inlets on the ceiling spread evenly on the floor and discharged smoothly through 20 ventilation outlets installed in the floor, greatly reducing the size of the area in which the air in the classroom was stagnant. From this, the average age of air in the entire classroom space was 15% lower than that in the reference classroom, and the difference in age of air between the front and rear areas of the classroom was greatly reduced to 1.5%. In conclusion, it was confirmed that in the classroom to which the vertical airflow ventilation scheme was applied, indoor air quality was substantially improved, and the air quality deviation was small compared to those in the classroom to which the conventional ventilation scheme was applied. Therefore, by applying the vertical airflow ventilation scheme devised in this study to a classroom, the air quality in the classroom can be greatly improved and a uniform air quality environment can be provided regardless of the location of the students, which will greatly help improve students’ health and learning ability.

## Data Availability

Not applicable.
